# Unexplained intraoperative cardiac tamponade during thoracoscopic lobectomy: a case report

**DOI:** 10.3389/fsurg.2026.1936557

**Published:** 2026-09-30

**Authors:** Wenyu Zhang, Ruixu Yan, Xiujie Lu, Junchao Liu

**Affiliations:** Department of Anesthesiology and Surgery, Qingdao Municipal Hospital, Qingdao, Shandong, China

**Keywords:** cardiac tamponade, echocardiography, pericardiotomy, pneumonectomy, thoracic surgery, video-assisted

## Abstract

**Background:**

Cardiac tamponade is a life-threatening emergency characterized by reduced cardiac output and circulatory failure. It most commonly occurs during cardiac surgery, interventional cardiology procedures, pericardiocentesis, or central venous catheterization, as well as in the setting of acute myocardial infarction or aortic dissection. To the best of our knowledge, cardiac tamponade during video-assisted thoracoscopic surgery (VATS) lobectomy has not been reported and may has an insidious presentation, making diagnosis challenging in the absence of obvious pericardial injury.

**Case presentation:**

We report a case of unexplained intraoperative cardiac tamponade in a 74-year-old man undergoing VATS left upper lobectomy. During hilar vessel dissection, the blood pressure decreased to 82/65mmHg. The heart rate increased to 95 beats/min, requiring norepinephrine infusion (0.2–0.3 μg·kg⁻¹·min⁻¹) to maintain blood pressure. However, no bleeding was evident in the surgical field, hemoglobin and lactate levels remained stable, and oxygenation and airway pressures were unchanged. After considering and excluding major differential diagnoses, including tension pneumothorax, hypovolemic shock, and pulmonary embolism, intraoperative point-of-care transthoracic echocardiography (TTE) was performed and revealed pericardial effusion with echocardiographic features of cardiac tamponade. Creation of a pericardial window with drainage of approximately 200 mL of bloody fluid. Immediately, blood pressure rose to 170/90 mmHg and heart rate decreased to 82 beats per minute. No definitive source of bleeding was identified during the operation. On postoperative day1, the patient developed paroxysmal atrial fibrillation, which resolved after treatment with amiodarone. Serial TTE on postoperative days 1, 2, and 6 showed no significant abnormalities. The chest tube was removed on postoperative day 6, and the patient was discharged the following day.

**Conclusion:**

During VATS lobectomy, intraoperative hypotension that cannot be explained by blood loss or changes in ventilation parameters, particularly when accompanied by a narrowed pulse pressure, should prompt consideration of occult cardiac tamponade. Early point-of-care ultrasonography and timely pericardial drainage are essential for rapid diagnosis and hemodynamic stabilization.

## Introduction

VATS has become the mainstream approach for lobectomy owing to its advantages of minimal surgical trauma, faster postoperative recovery, and lower complication rates (1, 2). The classical clinical signs of cardiac tamponade accompany with VATS lobectomy may be attenuated or masked by general anaesthesia, positive-pressure ventilation, and patient positioning. As a result, recognition may be delayed while more common causes of hypotension are initially investigated. We present a case of sudden, refractory hypotension during a VATS left upper lobectomy, where point-of-care TTE performed by the anesthesiologist was pivotal in diagnosing cardiac tamponade secondary to a massive hemorrhagic pericardial effusion. The definitive surgical intervention, while lifesaving, did not identify a clear source of bleeding. We aim to: (1) describe and draw attention to this scarcely reported entity of spontaneous pericardial bleeding during pulmonary resection, and discuss its potential mechanisms and management implications; (2) emphasise the indispensable role of anesthesiologist-performed intraoperative echocardiography in the rapid differential diagnosis of acute haemodynamic collapse.

## Case presentation

A 74-year-old male (height: 173 cm; weight: 59 kg) planned a thoracoscopic left upper lobectomy for a space-occupying lesion in the left upper lobe of the lung. He had no notable past medical history and any history of anticoagulant medications. Preoperative laboratory tests, pulmonary function tests, and electrocardiograms all showed no significant abnormalities. TTE demonstrated aortic valve degeneration with mild tricuspid and mitral regurgitation (Figure 1A). Pulmonary function tests revealed no surgical contraindications. Preoperative chest CT identified a space-occupying lesion in the left upper lobe (Figure 2A), and pathological results suggested carcinoma. Preoperative assessment confirmed that his cardiopulmonary function was adequate to tolerate surgery.

**Figure 1:**
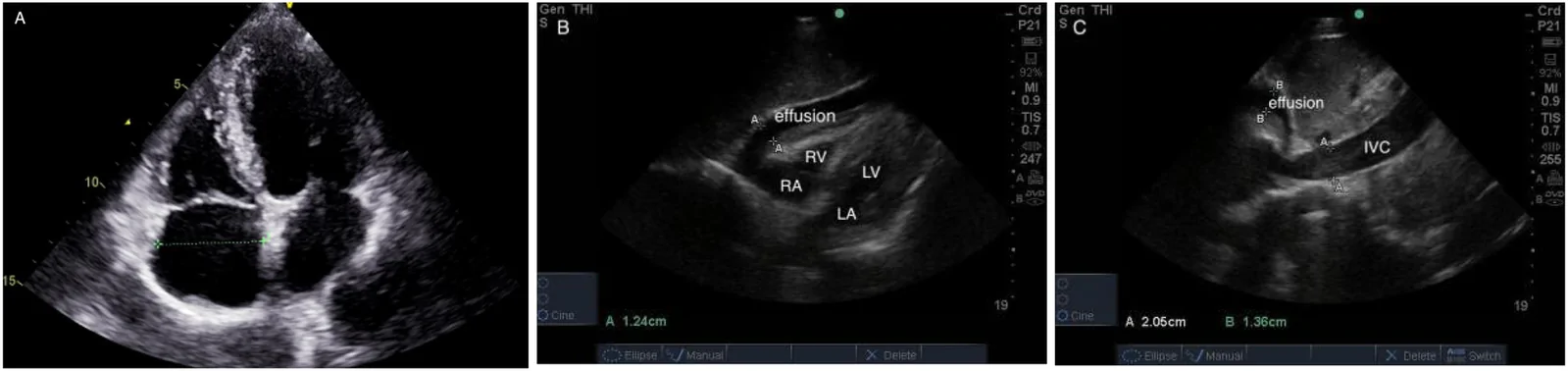
**(A)** Preoperative echocardiogram showing no pericardial effusion. **(B)** Subxiphoid echocardiographic view revealing an anechoic area (effusion) adjacent to the right ventricle, lateral to the right atrium, and at the apex, consistent with cardiac tamponade. **(C)** Ultrasound showing a dilated inferior vena cava with less than 50% inspiratory collapse.

**Figure 2:**
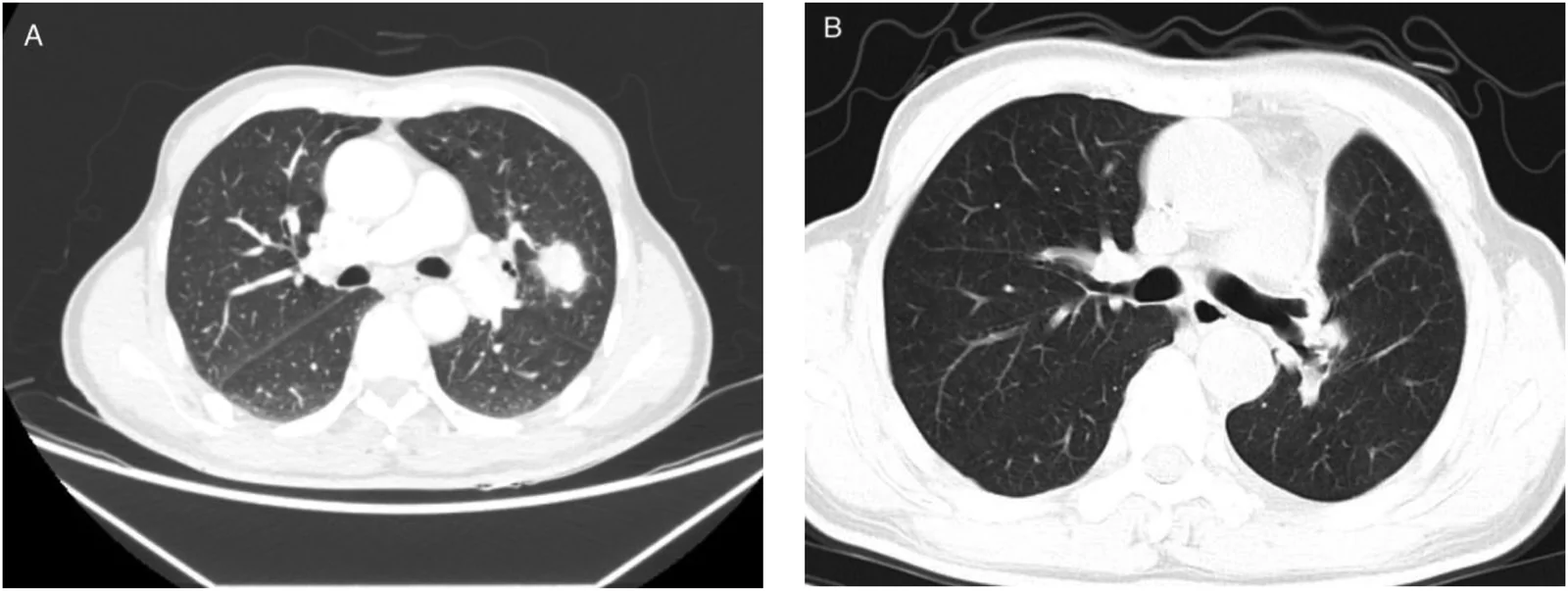
**(A)** Preoperative CT showing left upper lobe space-occupying lesion, highly suggestive of neoplasm. **(B)** Four-month follow-up postoperative CT after left upper lobectomy.

On the day of surgery, the patient entered the operating room at 07:45. Initial non-invasive monitoring showed a blood pressure (Bp) of 122/85 mmHg, heart rate(HR) of 85 bpm, and SpO₂of 98%. Ultrasound-guided radial artery cannulation for invasive monitoring showed a Bp of approximately 140/80 mmHg. Routine anesthetic induction was performed at 08:15. A 37-Fr right-sided double-lumen endotracheal tube was inserted, and correct positioning was confirmed using fiberoptic bronchoscopy. At 8:35, the patient was positioned in the right lateral decubitus position, and an ultrasound-guided intercostal nerve block was performed. Surgery commenced at 8:45 with one-lung ventilation of the right lung, and the airway pressure was 26 cmH₂O.

### Intraoperative hemodynamic collapse: investigation and management

Approximately 70 min after surgical incision, at 9:55, while the surgeon was dissecting branches of the left upper lobe pulmonary artery, the patient's blood pressure abruptly decreased from 120/65 mmHg to 82/65 mmHg. The heart rate increased from 60 to 95 beats/min, while sinus rhythm was maintained without arrhythmia. The invasive arterial pulse pressure narrowed from a preoperative baseline of 40–50 mmHg to 15–20 mmHg. SpO₂ remained at 100%, and airway pressure during one-lung ventilation remained unchanged. The anesthesiologist discussed the possible cause of hypotension with the surgeon. The surgeon reported a clear surgical field without active bleeding and confirmed that no surgical manipulation had compressed the major intrathoracic vessels or heart. Physical examination by the anesthesiologist revealed clear breath sounds over the right lung and no jugular venous distension. Because the left hemithorax was air-filled, cardiac auscultation was limited to the aortic area at the right sternal border, revealing a regular rhythm, tachycardia, and no abnormal heart sounds. Monitoring showed an end-tidal carbon dioxide (PetCO₂) level of 32 mmHg, a heart rate of 95–109 beats/min, and a peak airway pressure of 24–27 cmH₂O, without significant deviation from baseline values. Arterial blood gas analysis showed no significant changes in hemoglobin or lactate levels.

The anesthesiologist immediately administered a norepinephrine bolus, followed by continuous infusion at 0.2–0.3 μg·kg⁻¹·min⁻¹, maintaining blood pressure at approximately 90/73 mmHg. At 12:00, the left upper lobectomy was successfully completed. Repeat arterial blood gas analysis again showed no significant changes in hemoglobin or lactate levels. Given the absence of surgical bleeding and the stable hemoglobin level, hemorrhagic shock was considered unlikely. The absence of rash or fever also made distributive shock less likely. Unchanged airway pressure, oxygenation, and end-tidal CO₂ made tension pneumothorax and pulmonary embolism unlikely.

The surgeon reported that no surgical manipulation had compressed the major intrathoracic vessels or heart. In addition, pericardial exploration revealed no gross injury, and jugular venous distension was not remarkable. Blood pressure was maintainable with high dose norepinephrine at 0.2 μg/kg/min. Therefore, the anesthesiologist did not request surgical interruption for further investigation of the etiology of hypotension. The details of anesthesia and the lobectomy procedure are presented in Table 1.

**Table 1:** Anesthesia induction, maintenance, surgical procedure, and postoperative outcomes.

Time point	Clinical event
3 days prior to surgery	Underwent CT-guided percutaneous transthoracic needle lung biopsy; histopathological examination revealed findings suspicious for malignancy.
Day of surgery 7:45	Admitted to operating room; baseline hemodynamic parameters were within normal limits.
8:15	Induction of general anesthesia performed.
8:35	Patient positioned in lateral decubitus position; ultrasound-guided intercostal nerve block administered
8:45	Surgical procedure initiated.
9:55	Acute hypotensive episode occurred with narrowing of pulse pressure (from 30–40 mmHg to 15–20 mmHg); continuous intravenous infusion of norepinephrine was commenced.
10:00-12:00	Pulmonary lobectomy completed; norepinephrine infusion maintained at a rate of 0.2 µg/kg/min; serial monitoring revealed no significant fluctuations in hemoglobin concentration or serum lactate levels.
Post-12:00	Bedside transthoracic echocardiography (TTE) was performed, which confirmed the diagnosis of cardiac tamponade.
Approximately 13:00	Repeated video-assisted thoracoscopic surgery (VATS) was undertaken for pericardial fenestration and drainage; approximately 200 mL of hemorrhagic effusion was evacuated, followed by prompt hemodynamic restoration.
Postoperative day 1	New-onset atrial fibrillation was documented and subsequently controlled with antiarrhythmic pharmacotherapy.
Postoperative day 6	Thoracic drainage tube was removed.
Postoperative day 7	Patient was discharged from hospital.
4 months postoperatively	Follow-up chest CT demonstrated satisfactory recovery with no evidence of recurrence or complications.

### Diagnosis and secondary intervention

After completion of the thoracoscopic lobectomy, bedside TTE was performed by the anesthesiologist to determine the cause of hypotension. The heart could only be visualized through the subxiphoid window. TTE demonstrated hypoechoic areas in the pericardial cavity, primarily adjacent to the right ventricle, right atrium, and cardiac apex, findings consistent with cardiac tamponade (Figure 1B, subxiphoid view). The inferior vena cava measured approximately 2. 05 cm with inspiratory collapse of less than 50% (Figure 1C). Due to the unclear etiology of cardiac tamponade, both ultrasound and cardiac surgery consultations were sought, and a decision was made to proceed with thoracoscopic pericardiotomy to investigate the cause. Access was obtained through the original lobectomy incision, and the pericardium appeared tense and bulging (Figure 3A). A 1. 5 cm diameter fenestration was created in the left lateral pericardial wall, 3 cm posterior to the phrenic nerve (Figure 3B). Immediately upon opening, bright red blood and small clots were released, with a total of approximately 200 mL of hemorrhagic fluid and small clots drained. No source of active bleeding was identified, and the cause remained unclear. The pericardial cavity was irrigated with normal saline until the effluent was clear. After 20 min of observation with no further hemorrhagic drainage, routine chest closure was performed. Immediately following the pericardial window procedure, blood pressure rose to 170/90 mmHg and heart rate decreased to 90 beats per minute. After discontinuation of vasoactive agents, blood pressure stabilized at 110/70 mmHg with a heart rate of 80 beats per minute.

**Figure 3:**
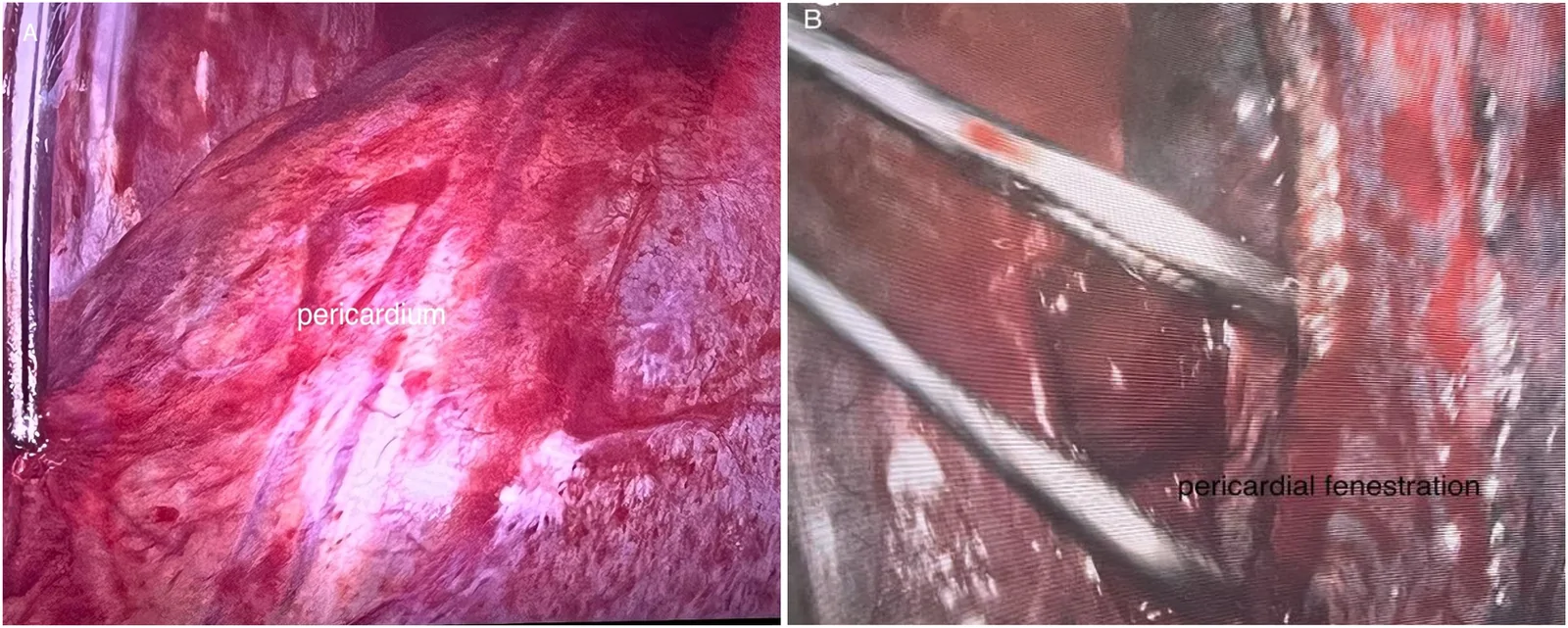
**(A)** Thoracoscopic view depicting a tense, bulging pericardium. **(B)** Site of pericardiotomy (∼1.5 cm). The pericardium is collapsed following drainage.

### Postoperative outcomes

Postoperative Day 1: Paroxysmal atrial fibrillation developed and was treated with intravenous amiodarone. Chest radiography showed a small residual left-sided pneumothorax, but the patient had no dyspnea or chest discomfort. Transthoracic echocardiography demonstrated preserved cardiac function. Pleural drainage yielded 330 mL of serosanguineous fluid.

Postoperative Day 6: The cardiac rhythm converted spontaneously to sinus rhythm, and repeat echocardiography showed no abnormality. Drainage had decreased to 190 mL, and the fluid had become pale yellow and serous. The chest tube was then removed.

Postoperative Day 7: The patient was discharged without complications.

Four-month postoperative follow-up: Chest computed tomography showed satisfactory re-expansion of the lung, with no evidence of late complications. The patient had returned to his usual daily activities without functional limitations.

### Case review and discussion

The occurrence of a spontaneous, non-traumatic, and source-occult pericardial hemorrhage during an otherwise routine elective VATS lobectomy, particularly in the absence of identifiable surgical trauma or underlying coagulopathy, has not been reported; however, postoperative cardiac tamponade following lobectomy has been documented in the literature, occurring from minutes up to over ten days postoperatively, with a predilection for left-sided lobectomy (3). Compared with previously reported cases of cardiac tamponade accompanied with VATS lobectomy, The underlying mechanisms of pericardial injury differed across the reported cases. In the present patient, the exact source of bleeding could not be determined. The pericardial effusion resolved after a pericardial window was created, and no additional intervention was required. In other reports, the cause was more readily identified. These causes included coronary artery laceration from a protruding staple line (4, 5), direct laceration of the ventricular wall by malformed staples (6, 7), rupture of an aberrant bronchial artery (8, 9), and unexplained postoperative cardiac tamponade (10, 11), as it did in our patient. In most reported cases. This case is unusual in that the tamponade developed intraoperatively during hilar dissection, symptoms appeared during the early postoperative period, from several hours to as long as 9 days after surgery. Although a few cases also had a very early onset, later presentation was more common overall.

Point-of-care TTE played an important role in the diagnosis. It was also useful in several of the previously reported cases (8–11), while others were diagnosed by computed tomography (CT) (4–6) or surgical re-exploration (7). In our patient, the atrial fibrillation was transient, and the subsequent recovery was uneventful, in keeping with the outcomes reported in several other cases. The treatment approaches were broadly similar: all patients underwent either creation of a pericardial window or pericardiocentesis, followed by immediate hemodynamic improvement. Part of patients had a more complicated course and required direct suture repair of ventricular lacerations or re-thoracotomy for coronary artery rupture (4, 5). This improvement did not eliminate the risk of a poor outcome, however; one patient subsequently died of septic shock (4).

Taken together, these cases show that cardiac tamponade after lung resection, although rare, can differ substantially in its timing, cause, and clinical severity. The present case emphasizes the importance of considering cardiac tamponade when unexplained intraoperative hypotension occurs despite the absence of obvious surgical bleeding. Immediate echocardiographic assessment can be decisive in recognizing the problem and allowing prompt decompression.

In retrospect, the marked narrowing of pulse pressure accompanying the onset of hypotension represented a critical early hemodynamic clue to cardiac tamponade, reflecting reduced stroke volume and cardiac output. At that time, switching electrocardiographic leads to assess for electrical alternans involving the QRS complexes, *P* waves, and T waves, together with careful evaluation of the invasive arterial waveform for variations in amplitude and changes in the dicrotic notch that may indicate pericardial compression, could have provided additional diagnostic clues. Such supportive findings might have prompted earlier bedside TTE or transesophageal echocardiography (TEE). Unfortunately, these signs were not sufficiently recognized at the time, resulting in delayed diagnosis and treatment. Furthermore, stable hemoglobin levels suggested a nonhemorrhagic cause of the hemodynamic deterioration and favored cardiogenic or obstructive shock. Notably, the abrupt decrease in blood pressure coincided with dissection of the left upper lobe pulmonary artery, which lies adjacent to the pericardium, raising the possibility of occult blunt injury associated with surgical manipulation.

We propose the following mechanism for this case: occult blunt injury to the pericardium or ventricular wall during surgical manipulation resulted in intrapericardial bleeding. As intrapericardial pressure progressively increased, compression of the bleeding site may have promoted spontaneous hemostasis and local clot formation (12). This hypothesis explains the absence of active bleeding after creation of the pericardial window and is consistent with several previously reported cases (6).

Currently, treatment options for cardiac tamponade include ultrasound-guided pericardiocentesis and surgical pericardiotomy, with ongoing debate regarding the optimal approach (13, 14). In this case, however, transthoracic ultrasound visualization was suboptimal following thoracoscopic lobectomy, making ultrasound-guided pericardiocentesis technically challenging. Because the patient remained under anesthesia, thoracoscopic access to the pericardium via the original approach was readily achievable, and cardiac surgeons were available to manage any potential cardiac or vascular complications.

### Educational takeaways

1. During VATS left upper lobectomy, surgical manipulation near the pericardium should be performed cautiously to minimize the risk of occult injury caused by blunt dissection or excessive traction.
2. In cases of intraoperative hypotension of unclear etiology, the absence of visible surgical bleeding together with stable hemoglobin levels should prompt consideration of causes beyond anesthetic depth or hemorrhage, including cardiac tamponade.
3. A narrowed pulse pressure is a readily detectable and potentially sensitive early hemodynamic sign of cardiac tamponade and should be closely monitored by the anesthesiologist.
4. When intraoperative hemodynamic changes cannot be explained by surgical manipulation or laboratory findings, point-of-care ultrasonography should be performed as early as possible. TEE is preferred when available; otherwise, prompt TTE examination should be performed. Diagnostic evaluation should not be deferred solely because adequate circulation can still be maintained.

## Data Availability

The raw data supporting the conclusions of this article will be made available by the authors, without undue reservation.
